# Anthocyanin Composition and Content in Rye Plants with Different Grain Color

**DOI:** 10.3390/molecules23040948

**Published:** 2018-04-19

**Authors:** Pavel A. Zykin, Elena A. Andreeva, Anna N. Lykholay, Natalia V. Tsvetkova, Anatoly V. Voylokov

**Affiliations:** 1 Faculty of Biology, St. Petersburg State University, Universiteskaya nab.7/9, St. Petersburg 199034, Russia; pavel.zykin@gmail.com (P.A.Z.); lankira@mail.ru (A.N.L.); ntsvetkova@mail.ru (N.V.T.); 2 Laboratory of Plant Genetics and Biotechnology, Vavilov Institute of General Genetics Russian Academy of Sciences, St. Petersburg branch, Universiteskaya nab. 7/9, St. Petersburg 199034, Russia; av_voylokov@mail.ru

**Keywords:** rye inbred lines, genes of grain color, anthocyanin identification, comparative genetics, HPLC-MS analysis

## Abstract

The color of grain in cereals is determined mainly by anthocyanin pigments. A large level of genetic diversity for anthocyanin content and composition in the grain of different species was observed. In rye, recessive mutations in six genes (vi1...vi6) lead to the absence of anthocyanins in all parts of the plant. Moreover, dominant genes of anthocyanin synthesis in aleurone (gene C) and pericarp (gene Vs) also affect the color of the grain. Reverse phase high-performance liquid chromatography and mass spectrometry were used to study anthocyanins in 24 rye samples. A lack of anthocyanins in the lines with yellow and brown grain was determined. Delphinidin rutinoside and cyanidin rutinoside were found in the green-seeded lines. Six samples with violet grains significantly varied in terms of anthocyanin composition and content. However, the main aglycone was cyanidin or peonidin in all of them. Monosaccharide glucose and disaccharide rutinose served as the glycoside units. Violet-seeded accession forms differ in the ratio of the main anthocyanins and the range of their acylated derivatives. The acyl groups were presented mainly by radicals of malonic and sinapic acids. For the colored forms, a profile of the revealed anthocyanins with the indication of their contents was given. The obtained results are discussed in connection to similar data in rice, barley, and wheat, which will provide a perspective for future investigations.

## 1. Introduction

Due to the peculiarities of their chemical structure, anthocyanins have certain biological activity, which manifests itself in the composition of plant food. In cereals, the edible part is the grain (caryopsis). The grain is a single-seeded fruit, the tissues of which accumulate specific metabolites, including anthocyanins. Anthocyanins and the structurally similar proanthocyanidins are one of the end products of the branched chain of flavonoid biosynthesis [1]. Their accumulation in the caryopsis of cereals occurs in the cells of the fruit (pericarp) and seed (testa) coats as well as in the aleurone layer of the endosperm. The synthesis of anthocyanins and their intra- and intercellular movement is controlled by structural and regulatory genes [1]. The mutational variability of these genes leads to a change in the qualitative and quantitative composition of anthocyanins in grain, which is largely influenced by environmental conditions. During the course of domestication and further breeding, the composition of metabolites, including anthocyanins, significantly changed in cereal grains [2]. This has led to most modern varieties of wheat, barley, maize, and rice being represented by genotypes that are not capable of synthesizing anthocyanins in the grain. Currently, taking into account the aim to improve health through grain nutrients, work has been conducted to recreate the genotypes that can accumulate anthocyanins and their precursors in the grain of cereals [3]. The source of colorful genes for cereal improvement include local varieties, genetic collections, and wild relatives. For the accumulation of anthocyanins, genetic engineering approaches have also been developed [1]. The composition and concentration of anthocyanins in small cereals largely reflect the color of the grain. Wheat and barley produce yellow (white), blue (green), red (brown), and purple (violet, black) grains. The cultural and weedy rye revealed the same range of variation in coloration of grain. This implies a similar genetic control and approximately the same composition of anthocyanins in all the cereals with variously colored grain. The first data on the composition of anthocyanins in small cereals were obtained in rye. The analysis of anthocyanin pigments in green [4] and purple (violet) [5] grain was carried out using paper chromatography. This established that green grain contains delphinidin 3-*O*-rutinoside as the main pigment. In the purple grain, the composition of anthocyanins was richer. Cyanidin 3-*O*-glucoside and peonidin 3-*O*-glucoside in addition to trace amounts of cyanidin 3-*O*-rutinoside and peonidin 3-*O*-rutinoside were found. In addition, acylated forms were found for all four glycosides, but the composition of acyl residues was not established. Similar results on other cereals were subsequently obtained [6]. The improvement of separation and identification techniques has significantly expanded the understanding of anthocyanin composition in related cereals [7,8,9], although this has not occurred in rye. The purpose of this paper is to fill this gap and to discuss new data on anthocyanin composition in related species of wheat, barley and rice.

## 2. Results

**Figure 1:**
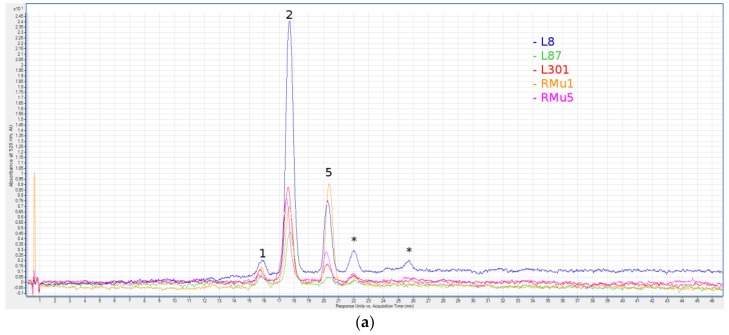

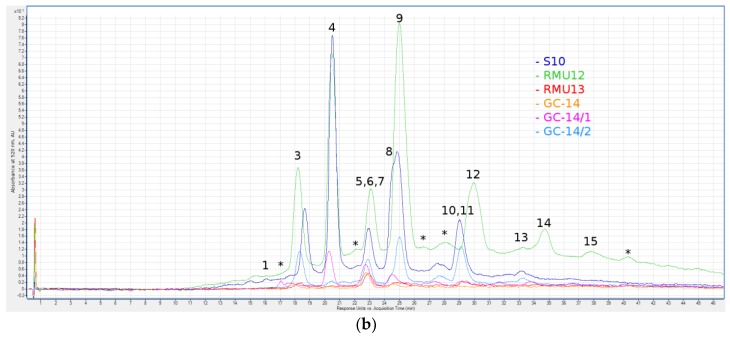
Liquid chromatography with spectrophotometric absorption detection in visible spectrum (LC-VIS) chromatograms at 520 ± 4 nm of green grain (**a**) and violet grain (**b**) seeds extracts. 1—Delphinidin 3-*O*-Glucoside; 2—Delphinidin Rutinoside; 3—Cyanidin 3-*O*-Glucoside; 4—Cyanidin Rutinoside; 5—Pelargonidin Rutinoside; 6—Malvidin 3-*O*-Galactoside; 7—Peonidin 3-*O*-Glucoside; 8—Peonidin Rutinoside; 9—Cyanidin (malonyl)hexoside; 10—Peonidin (malonyl)hexoside; 11—Cyanidin (dimalonyl)hexoside; 12—Cyanidin (succinyl)hexoside; 13—Peonidin (dimalonyl)hexoside; 14—Unidentified Peonidin; 15—Cyanidin (sinapoyl)hexoside; and *—peaks for which no anthocyanin aglycone was identified.

The high-performance liquid chromatography-mass spectrometry (HPLC-MS) analysis of 24 rye samples with different grain colors showed an absence of a detectable level of anthocyanins in ten accessions with yellow grain and in three accessions with brown grain. A uniform pattern was established for the five lines of independent origin, which is described as green-seeded (Table 1, Figure 1a). Delphinidin rutinoside was found in all five lines, cyanidin rutinoside was detected in four of them and delphinidin 3-*O*-glucoside was found in one line (L301). A specific feature of rye as a cross-pollinated species is inbred depression, which is manifested as a sharp decrease for all quantitative traits of plants during inbred propagation. The amount of anthocyanins is not an exception. All studied inbred lines with green and violet (GC-14/1, GC-14/2) seed colors have low anthocyanin content, which does not exceed 1 mg/kg (Table 1).

Through a decrease in the total content of anthocyanins, inbred depression can have a non-specific effect on the composition of detected anthocyanins, especially on the detection of minor components. Between the three related samples (GC-14, GC-14/1 and GC-14/2), differences in the composition of the minor anthocyanins were found (Table 1, Figure 1b). Peonidin 3-*O*-glucoside was found in all three forms. In GC-14/1, only this anthocyanin was found, while additional peonidin derivatives were also found in the original sample of GC-14, which were namely peonidin 3-*O*-glucoside and peonidin (malonyl)hexoside. GC-14 was distinguished by the presence of malvidin 3-*O*-galactoside, which was not found in other violet forms. Differences, such as the aglycone (malvidin) and glycosyl residue (galactoside), represent the genetic heterogeneity of GC-14, which has been reproduced from the moment of its isolation by cross-pollination of plants within the plot. GC-14/2 is distinguished from both related forms by the presence of two cyanidin derivatives: cyanidin rutinoside (0.10 mg/kg); and cyanidin 3-*O*-glucoside detected at the limit of sensitivity. The inbred lines RMu12 and RMu13 are similar in the expression of intense anthocyanin coloration of all parts of plants, including grains. The similarity of line genotypes was indicated by the very close profile of anthocyanins found in these forms (Table 1, Figure 1). In both lines, the rutinosides of cyanidin and peonidin dominated, while lower concentrations of glucosides of both anthocyanidins and their derivatives with one or two residues of malonic acid were found. The composition of anthocyanins in the grain of these lines was distinguished by the presence of a significant concentration of another anthocyanin with an aliphatic acyl group in RMu12, which was namely cyanidin (succinyl)hexoside. Furthermore, another distinguishing characteristic is the presence of pelargonidin rutinoside as a minor component in RMu13. In all three lines with a high concentration of anthocyanins (S10, the RMu12 and RMu13) anthocyanin-containing acyl groups of an aromatic sinapic acid was found namely, cyanidin (sinapoyl)hexoside. Line S10 differs by the high content of glycosides attached to cyanidin and peonidin cores, added by one or two residues of malonic acid. Moreover, the predominant glycoside residue in line S10 is monosaccharide glucose, while disaccharide rutinose is more common for the violet-seeded lines RMu12 and RMu13.

To provide an alternative quantitative approach, we measured the concentration of anthocyanins in the anthocyanin-rich forms of S10, RMu13 and RMu12 by the spectrophotometric pH–differential method. The results and data used for calculations are summarized in Table 2. 

The variations in data between the two methods used could be explained by the use of the spectrophotometric method only for the major anthocyanin, despite the use of complex mixtures; and the use of cyanidin (λ_vis-max_ = 516) equivalents of anthocyanins for HPLC-MS due to the lack of other quantitative standards. Nevertheless, the difference is small and consistent, allowing us to conclude that the line RM12 is exceptionally high in anthocyanin content. The total anthocyanin content calculated using both methods is on average 15 times more in the RMu12 than in the S10, which is significantly higher than the other three violet-seeded accessions that only contain 0.11–1.63 mg/kg.

## 3. Discussion

The synthesis of anthocyanin pigments in grain of cereals is localized in the maternal tissues—fruit (pericarp) and seed (testa) coats as well as in the hybrid tissue—aleurone layer of the endosperm. Each of these tissues is characterized by a certain composition of anthocyanins and related compounds. The qualitative and quantitative composition of pigments is controlled by structural and regulatory genes (transcription factors). Along with similarity in the genetic control of anthocyanins in all cereals, the species specificity and significant intraspecific variability due to non-lethality of mutations in genes of anthocyanin biosynthesis were established [10]. Dominant alleles of regulatory genes coloring aleurone and pericarp in bread wheat were introgressed in its genome on the basis of distant hybridization [11]. The native genes are only homeological loci (R1–R3) of the red coloration of the testa, which controls three MYB-type transcription factors [12]. In barley, the variability for grain color exists due to the presence of many spontaneous and induced mutants [13]. In rice, this variability is due to the diversity of grain color in local varieties. Open-pollinated rye varieties are heterozygous and heterogeneous populations, due to the action of a rigid system of gametophytic incompatibility. They are also heterogeneous in terms of the color of the grains. In most varieties, there are green, yellow and rarely brown grains, between which there are grains of intermediate colors. The purple (violet) grain is typical for some samples of weedy rye [14]. The use of self-compatible (self-fertile) mutants allows differentiation of populations for inbred lines and to fix mutant alleles of genes in them, including those for grain color [14]. No anthocyanins were detected in brown-seeded and yellow-seeded lines, including those without anthocyanin. This corresponds to the absence or very low content of anthocyanins in white, red and brown rice grain; yellow grain in barley; and yellow and red grain in bread wheat [6]. The absence or low content of anthocyanins in all layers of the grain explains their yellow color in all four species. The red color of caryopsis in rice and wheat is associated with the color of testa. The color of testa in rice and wheat grain, as well as the brown color of the pericarp in certain genotypes of rice, is explained by the synthesis of proanthocyanidins—oligomeric and polymeric compounds related to leucoanthocyanidins, which are colorless precursors of colored anthocyanidins. In barley, proanthocyanidins (procyanidin B3, prodelphynidin B3) and their precursor (+)-catechin were detected well before other small cereals [13]. In the grains of barley, they do not have any color. Oligomers and polymers of proanthocyanidins and their precursors flavan-3-ol units are detected by chemical agents (solution of vanillin in hydrochloric acid). The red color of the grain in wheat is explained by the accumulation in the testa of closely related flavonoid compounds, which are converted into a red-brown insoluble pigment as the kernels mature [15]. It was established that the accumulation in the testa of proanthocyanidins and their precursors in barley and wheat control the orthologous MYB-type transcription factors Hvmyb10 and Tamyb10, respectively [12]. In rye, the variability in the color of testa was not revealed as the grain of the three main colors have brown testa. We have shown that the testa in rye reacts with reagents for proanthocyanidins and their precursors (unpublished). In the presence of vanillin, the brown color of the testa changes to red and when processed with the 4-(Dimethylamino)-cinnamaldehyde (DMACA) solution, the testa becomes blue. However, the nature of the pigment in rye grain with a superficial brown color, which is characteristic of the samples studied by us, has not been established. A previous study [16] showed that the brown color of grain in the line GK-37 arises from the interaction of two genes, with the brown grain producing plants with vi1 vi1, VsVs genotype. A similar interaction of the anthocyaninless mutation (rd) and the dominant allele (Rc–) of the purple grain coloring gene was established in rice [17]. The complementary Rd and Rc rice genes control the structure of dihydroflavonol-4-reductase and MYB-type transcription factor, respectively. As a result of the action of dihydroflavonol-4-reductase leucoanthocyanidins, the precursors of both anthocyanins and proanthocyanidins are formed. It is assumed that the formation of proanthocyanidins in brown pericarp is a consequence of incomplete loss of enzyme activity. The resulting intermediates accumulate in the genotypes of rd rd, Rc– and create a brown pigment. The function of genes vi1 and Vs in rye is unknown.

The green color of the grain in rye and the blue in bread, wheat, and barley are associated with the synthesis of anthocyanins in the aleurone layer of the endosperm. Data on the presence of anthocyanins in aleurone of rice caryopsis are absent in the available literature. Genes of blue aleurone in bread wheat are transferred to its genome, which is composed of whole chromosomes of the fourth homological group or their fragments from related wild species [11]. In barley, a combination of dominant genes (alleles) controlling three types of transcription factors [18] is responsible for the synthesis of anthocyanins in aleurone. The presence of anthocyanins in green grain in rye is associated with a dominant gene C. For the manifestation of the green coloring of rye grain, the presence of dominant alleles in all the loci vi1–vi6 [14,16] is obligatory. The synthesis of anthocyanins in pericarp of barley, wheat, and rice is controlled by corresponding transcription factors [19,20,21]. In rye, the gene Vs acting in a similar manner at the morphological level was identified and mapped [16]. The composition of anthocyanins in colored aleurone of barley, wheat, and rye is related to the major aglycone, which is delphinidin. Our data confirmed the previously obtained data [4] for green-seeded rye. Furthemore, detection of cyanidin rutinoside in addition to delphinidine rutinoside in green rye grain was done for the first time. Cyanidin derivatives typically dominate in the colored pericarp of barley, wheat, and rice [8,9]. 

In blue and purple barley, the derivatives of all six major anthocyanidins were identified, which are accumulated in different concentrations in aleurone or pericarp mainly in the form of monoglucosides. Two of them, which are namely the cyanidin 3-*O*-glucoside and peonidin 3-*O*-glucoside, are dominant over the others in the purple barley pericarp [6,7,22]. Only these two anthocyanins with total anthocyanin content of 52.1–1683.6 µg/kg were detected in rice, which has a grain color that varies from light purple to black [23]. Similar results were obtained by other researchers [24,25]. However, the review of 25 works reported 18 different anthocyanins identified in purple rice. Among them, only four could be quantified, which are two major anthocyanins, namely cyanidin-3-*O*-glucoside (51–84%) and peonidin-3-*O*-glucoside (6–16%); and two minor anthocyanins, which are cyanidin-rutinoside (3–5%) and cyanidin-3-*O*-galactoside (1–2%). The glycosides of other four main anthocyanidins (pelargonidin, delphinidin, petunidin and malvidin) besides cyanidin and peonidin were also discovered in some rice genotypes. These anthocyanins can prevail in some rare genotypes. Thus, in the cultivar Chinakuromai, petunidin 3-*O*-glucoside composed almost half of the anthocyanin content. It is interesting that all anthocyanins identified in purple (black) rice are comprised of mono- and disaccharides of six major anthocyanidins, although the acyl derivatives were not found [8]. 

For the first time, anthocyanins in purple grain were studied in rye [5]. Cyanidin 3-*O*-glucoside and peonidin 3-*O*-glucoside were found to be the main anthocyanins, while there were trace amounts of cyanidin rutinoside, peonidin rutinoside and acylated forms of these glycosides in five inbred lines with purple (violet) grain. The authors highlighted the variation in the ratio of aglycones and acylated derivatives in the studied lines of rye. Our data confirmed that the main aglycones in purple rye are cyanidin and peonidin, which is consistent with other studied cereals with purple grains. For the first time, acyl residues were identified in anthocyanins and their quantitative ratio was established in five rye forms with violet (purple) grains. Significant differences in the qualitative and quantitative composition of anthocyanins in violet-seeded lines were found. Self-fertility of the studied accessions allows us to select a constant sub-line with a high proportion of acylated anthocyanins and high total anthocyanin concentration. It can be used for segregation analysis of anthocyanins in hybrid progenies, with the objective of identifying the genes responsible for inter-line differences. The determination of molecular function of genes which were discovered at the morphological level is of great interest. The available data on candidate anthocyanin genes identified in other cereals can be effectively used through partial sequencing of the rye genome [26]. On the basis of such investigations, a new method will appear for constructing gene markers to obtain the different combinations of anthocyanin genes. For example, it is possible to produce double mutants for vi1–vi6 mutations using markers; to combine each of them with dominant C and Vs genes; and to study anthocyanins and their precursors in the constructed genotypes. Such new genotypes may be useful to resolve different problems, in particular to clarify the chemical nature of brown pigment in the rye grain and to describe the pleiotropic effect of regulatory genes on the metabolism in different kernel compartments. This will allow us to produce breeding material for production of rye varieties with health-promoting effects.

## 4. Materials and Methods

### 4.1. Plant Material

The composition of anthocyanins was analyzed in 24 forms and inbred lines of rye, which differed in terms of grain color and the presence of anthocyanins on other parts of the plant (Table 3). Every lacking anthocyanin form carries one of the six recessive mutation (vi1, vi2, vi3, vi4, vi5 or vi6) leading to the absence of anthocyanin coloration of coleoptile, nodes of the stem, glumes and awns of ears and grain. Segregation analysis revealed non-allelic nature of these mutations. The mutant genes have been designated by Latin name viridis (vi) [14] in accordance to the absence of anthocyanin on mutant seedlings, which are green for this reason.

The variety Esto (vi1) used in the work is a population consisting of self-incompatible genotypes. The accession of genetic collection GC-22 (vi2) was originally created by cross-pollination of plants without anthocyanin, which was selected in two inbred progenies. The form of GC-23 (vi3) was produced under self-pollination of the cloned plant of Vyatka variety. The forms of GC-149 (vi4), GC-151 (vi5) and GC-150 (vi6) were obtained on the basis of hybrids of three different plants of Vyatka variety with self-fertile lines. For many years, forms without anthocyanin were propagated by cross-pollination and their homozygosity was checked by the absence of anthocyanin coloration. Furthermore, for each accession, inbred lines were produced using single seed descent from the original plant. In the course of this work, from the accession GC-22, two sublines were produced that differed in color of kernels: line GC-22 yellow with yellow grains and line GC-22 brown with a light-brown grains. The yellow-seeded forms with anthocyanin color of coleoptiles, nodes and glumes were presented by the accession GC-12 and three highly-inbred lines of independent origin (L2, L5, L7). The inbred lines L2, L5 and L7 were the accessions from Peterhof genetic collection of rye. They originated from plants of three different rye varieties and reproduced by selfing for a long time. The uniformity of the yellow coloring of grain in the hybrids between these lines allows us to identify these lines as the recessive homozygote cc [14]. The dominant allele of this gene C leads to the green color of grain due to the presence of anthocyanins in aleurone. The green-seeded rye with this gene was represented by three highly-inbred lines (L87, L301, L8), line RMu5 and line RMu1 green, which was isolated from the sample of variety Esto. From the same sample, the inbred line RMu1 brown was isolated, which had a constant brown color of the grain. In the test crosses, it was shown that this line is homozygous for allele vi1, which causes the absence of anthocyanin color of plants in the variety Esto. Brown grain is a characteristic of line GC-37, which is derived from a hybrid between violet-seeded (gene Vs) and rye lacking anthocyanin (gene vi1). The genotype of this line, which is vi1vi1, VsVs=, causes the brown color of grain and the absence of anthocyanin color of the normally colored vegetative parts of the plant. Of the six violet-seeded samples (gene Vs), three are related. This is a sample of a weedy rye GC-14 and the two related inbred lines (GC-14/1 and GC-14/2), which were obtained from one initial plant of this population. RMu12 and RMu13 lines in addition to the purple color of the grain are characterized by highly colored red stems. The constant manifestation of these traits was observed in plants of three inbred generations. A grain sample of the spring purple rye S10, obtained from J. Sybenga (Agricultural University, Wageningen, The Netherlands), was heterogeneous and included a green and purple grain. Within two generations of inbred propagation, constant lines were obtained with green and purple grains. All forms with the designation RMu and line L301 have been kindly provided by P. Wehling (JKI, Groß Lüsewitz, Germany) and line L87 by A. Börner (IPK, Gatersleben, Germany). The other accessions and lines are original and are reproduced in the Peterhof genetic collection [14].

### 4.2. Sample Preparation and Chemicals

A total of 24 rye accessions with different grain colors harvested in 2016 and 2017 were selected for the analysis. For each accession, about 100 grains were weighted, vacuum-dried in a centrifugal vacuum concentrator (Labconco Centrivap) at room temperature until no further change in mass was observed. On average, drying to total dryness took 36 h. Dry mass was recorded for further reference. Grains were ground with household grain mill (Zepter MixSy) for 5 min. Standards of cyanidin chloride and malvidin 3-*O*-galactoside were from Sigma-Aldrich (St. Louis, MO, USA), HPLC-MS grade acetonitrile was purchased from Panreac (Barcelona, Spain), while HPLC-grade formic acid and other chemicals were from Vecton (St.-Petersburg, Russia). 

### 4.3. Extraction of Anthocyanins

The anthocyanins were extracted according to a previous study [27], with some modifications. We chose 70% ethanol acidified by 1% (*w*/*v*) citric acid as an extraction solvent to avoid esterification of the free carboxyl group of acylated anthocyanins and to prevent their deacylation. Furthermore, all subsequent steps up to HPLC-MS were carried out at a low temperature (<30 °C) for the same reason. 

The extraction was done with three portions of extraction solvent for 24 h at +4 °C in the dark. The supernatant was combined and dried to 3 mL on vacuum concentrator (Labconco Centrivap) at room temperature.

### 4.4. Anthocyanins Purification

**Figure 2:**
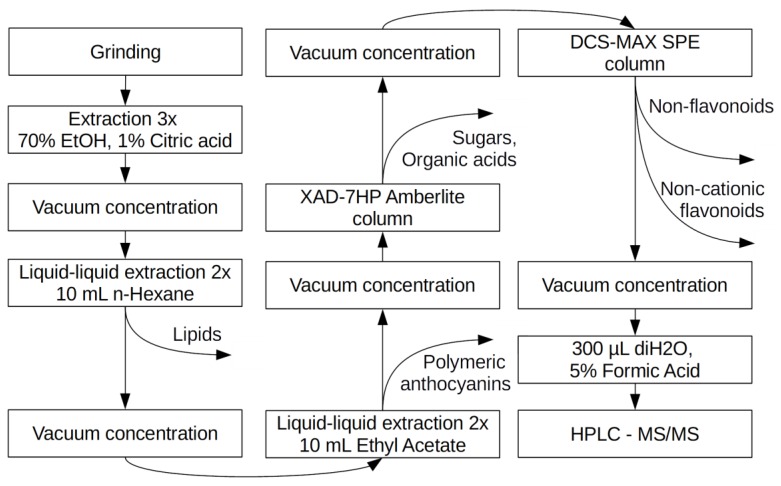
Stages of monomeric anthocyanins purification by selective removal of non-anthocyanin compounds.

The crude extract was purified by selective removal of non-anthocyanin compounds in stages (Figure 2).

Lipids were removed by liquid/liquid extraction with Hexane as follows, which was repeated twice. A total of 10 mL of hexane was added to 3 mL of extract, which was vortexed on BioSan V-32, Multi-Vortex for 15 min, centrifuged at 7000× *g* at +4 °C for 10 min with the water phase maintained. The solution was evaporated to dryness in the centrifugal vacuum concentrator and resuspended in 3 mL of deionized water. Other non-polar compounds, including the polymeric anthocyanidins (proanthocyanidins) were removed by liquid/liquid extraction with ethyl acetate twice as follows: 10 mL of water-impregnated ethyl acetate was added to 3 mL of extract, which was vortexed on BioSan V-32, Multi-Vortex for 15 min, centrifuged at 7000× *g* at +4 °C for 10 min with maintenance of the water phase. The solution was evaporated to dryness in the centrifugal vacuum concentrator and resuspended in 1.5 mL of deionized water with 0.1% of formic acid. To remove sugars and organic acids, the solution was loaded on self-made single-use columns loaded with 1.5 mL of Amberlite HAD-7HP resin, which was equilibrated with 5 mL of 0.1% of formic acid on deionized water. Columns were rinsed with 10 mL deionized water and eluted with 3 mL of methanol acidified with 0.1% formic acid. The solution was evaporated to dryness in centrifugal vacuum concentrator and resuspended in 1.5 mL of deionized water with 0.1% of formic acid. To separate positively charged compounds, such as anthocyanins from other flavonoids, samples were loaded on a DSC-MCAX SPE cartridge, which was pre-conditioned with 1.5 mL of methanol and equilibrated with 5 mL of water with 0.1% formic acid. The cartridge was rinsed with 3 mL water with 0.1% formic acid solution. The flavanol glycosides were eluted with 3 mL methanol, while the anthocyanins were eluted with 3 mL of a 50:50 solution of 5 mM bicarbonate-ammonium buffer at a pH of 6.0 with added methanol. The choice of buffer was dictated by its compatibility with ESI-MS. The solution was evaporated to dryness in centrifugal vacuum concentrator and resuspended with 300 μL of deionized water with 5% of formic acid in chromatography micro-vials.

### 4.5. Identification and Semi-Quantification of Anthocyanins by HPLC-ESI-MS

An Agilent 6538 quadrupole-TOF mass spectrometer (Agilent Technologies, Palo Alto, CA, USA) with Agilent 1200 series high-performance liquid chromatography was used. Chromatography was conducted on Agilent Zorbax SB-C18 column (1.8 μm; 1 × 150 mm) because this type of column could withstand a low pH of 1. We used gradient HPLC, solvent A of 5% formic acid in water (*v*/*v*) and solvent B of acetonitrile. The gradient elution program was used as follows: 0–3 min, 0% B; 3–5 min, 0–3% B; 5–55 min, 3–30% B; 55–60 min, 100% B. The column temperature was 55 °С, flow rate was 180 μL/min and injection volume was 5 μL. For the identification of anthocyanins, electrospray ionization (ESI) was operated in the positive mode with a mass range of 100–1500 (*m*/*z*), drying gas temperature of 350 °С, flow rate of 7.0 L/min, nebulizer pressure of 30 psig, capillary voltage of 3500 V, fragmentary voltage of 175 V and skimmer voltage of 65 V. The device was operated in automated tandem mode with an isolation window of 1.3 a.m.u., collision energy of 30 V, molecular ions selection in the range of 260–1500 Da, one linear and 3 MS/MS spectra were acquired every 2.1 s. Simultaneous spectrophotometric detection was conducted with Agilent 1200 Series G1365D MWD at 520 nm with a slit width of 8 nm, speed of 2.5 Hz and flow cell volume of 0.5 μL.

To prevent carry-over between probes, each probe was followed by blank injection (solvent “A”), which showed no detectable peaks. 

Due to the absence of standards for all identified anthocyanins, the blueberry extract containing 20 well-characterized peaks was used as a retention time and fragmentation pattern standard (Supplementary Material Table S1: The database of anthocyanine standards and theoretical values used for the identification in this study). 

For semi-quantitative analysis, quantification of the sample’s anthocyanin content was conducted using the external standard calibration curve, which was obtained by triplicate analysis of cyanine and malvidin-3-galactoside standards sequentially diluted in 5% formic acid at the concentration from 0.5 to 0.0001 mg/mL. The amount of anthocyanins in the samples was determined by the liquid chromatography-electrospray ionization mass spectrometry (LC-ESI-MS) method, the extracted ion peak areas of each anthocyanin were expressed as equivalents of cyanidin chloride. The limit of detection (LOD) and limit of quantification (LOQ) were set as concentrations that gave signal-to-noise ratio (SNR) of 3 and 10, respectively. Results with SNR that were lower than LOD were excluded from the table, while results with an SNR between LOD and LOQ, being non-quantitative, were marked as “<LOQ”.

The positive identification of anthocyanin was reported when: (i) molecular ion *m*/*z* and aglycon fragment were ±0.05 Da of that of standards, or in absence of standards, ±0.05 Da of that of theoretical mono-isotopic mass; (ii) the retention time was ±1 min of that for the standard.

The total amount of anthocyanins in high concentration samples was also determined by spectrophotometric pH–differential method according to a study of Giusti and Wrolstad [28]. Briefly, 20 μL of sample was mixed with 80 μL of 0.025 M potassium chloride buffer (pH of 1.0) and 20 μL of sample was mixed with 80 μL of 0.4 M sodium acetate buffer (pH of 4.5). The absorbance of the mixture was measured at two wavelengths: at the maximum absorbance of major anthocyanin, as determined by ESI-MS (λ_vis-max_); and at 700 nm using a UV–Vis spectrophotometer BioRad xMarkII. 

The corrected absorbance was calculated as:

(1) $$A=\left(A_{\lambda \mathrm{vis}-\max}^{\mathrm{pH}1.0}-A_{700}\right)-\left(A_{\lambda \mathrm{vis}-\max}^{\mathrm{pH}4.5}-A_{700}\right).$$

The concentration was calculated as:

(2) $${\mathrm{Conc}}_{\mathrm{mG}/L}=\frac{A\times \mathrm{MW}\times \mathrm{DF}\times 1000}{\varepsilon \times 1}$$

where *A* is corrected absorbance; MW is molecular weight of the major anthocyanin; DF is the dilution factor; and *ε* is the molar absorbance of the major anthocyanin. 

## 5. Conclusions

The improvement of the isolation procedures, the use of modern analytical methods and the involvement of diverse genetic material has allowed for the gathering of new data on the composition of anthocyanins in the colored grain of cereals. In pericarp and aleurone in wheat and barley as well as in pericarp of rice, anthocyanins derived from all six major anthocyanidins were identified: pelargonidin, cyanidin, peonidin, delphinidin, petunidin, and malvidin. In green and violet rye kernels the all of the anthocyanidins except for petunidin were identified. Anthocyanins differ in position, number, and type of glycosyl and acyl residues that are associated with an anthocyanid core. A common rule is the predominance of delphinidin as the major aglycone in blue (green in rye) grain and cyanidin derivatives in the purple (violet in rye) grain [9]. The so-called black color of grain may be the result of a very high concentration of anthocyanins in pericarp or its presence in pericarp and aleurone simultaneously. An additional rule is the accumulation of proanthocyanidins in the testa of all four discussed species. In certain genotypes of rice, the accumulation of proanthocyanidins is also established in the pericarp. Obviously, modification of the anthocyanin structure requires the presence of corresponding regulatory and structural genes in the plant genome. Mutational variability of these genes and influence of the environment lead to a wide variety of composition and concentration of anthocyanins in cereal grain

## Figures and Tables

**Table 1:** Identification and tentative quantification of anthocyanin composition in rye grain.

Anthocyanin ^1^	M/Z Molecular Ion	M/Z Fragment Ions	RT, min	Content mg/kg of Dry Matter ^3^
**Green grain L8, 0.18 mg/kg**				
**Delphinidin Rutinoside**	611.16	303.05, 465.11	17.8	0.15
Cyanidin Rutinoside	595.17	287.05, 449.11	20.3	0.03
**Green grain L87, 0.07 mg/kg**				
**Delphinidin Rutinoside**	611.16	303.05, 465.11	17.9	0.07
**Green grain L301, 0.88 mg/kg**				
**Delphinidin Rutinoside**	611.16	303.05, 465.11	17.9	0.88
Cyanidin Rutinoside	595.17	287.05, 449.11	20.4	<LOQ ^2^
Delphinidin 3-*O*-Glucoside	465.10	303.05	15.9	<LOQ
**Green grain RMu1 green, >0.0 mg/kg**				
**Cyanidin Rutinoside**	595.17	287.05, 449.11	20.4	<LOQ
Delphinidin Rutinoside	611.16	303.05, 465.11	17.8	<LOQ
**Green grain RMu5, >0.0 mg/kg**				
**Delphinidin Rutinoside**	611.16	303.05, 465.11	17.9	<LOQ
Cyanidin Rutinoside	595.17	287.05, 449.11	20.5	<LOQ
**Violet grain S10, 32.6 mg/kg**				
**Cyanidin (malonyl** **)hexoside)**	535.11	287.06	25.0	7.39
Cyanidin 3-*O*-Glucoside	449.11	287.06	18.7	6.47
Peonidin (malonyl)hexoside	549.12	301.07	29.3	6.03
Peonidin 3-*O*-Glucoside	463.12	301.07	22.9	5.23
Cyanidin (dimalonyl)hexoside	621.11	287.06	29.1	3.57
Peonidin (dimalonyl)hexoside	635.13	301.07	33.3	2.04
Cyanidin Rutinoside	595.17	287.05, 449.11	20.6	0.72
Peonidin Rutinoside	609.18	301.07, 463.12	24.6	0.72
Cyanidin (sinapoyl)hexoside	655.17	287.05	36.6	0.43
Cyanidin (malonyl)hexoside	535.11	287.05	25.1	<LOQ
**Violet grain RMu12, 403.75 mg/kg**				
**Peonidin Rutinoside**	609.18	301.07, 463.12	25.0	115.37
Cyanidin Rutinoside	595.17	287.05, 449.11	20.5	90.98
Cyanidin (succinyl)hexoside	549.12	287.06	30.1	55.27
Cyanidin 3-*O*-Glucoside	449.11	287.06	18.2	40.10
Cyanidin (malonyl)hexoside	535.11	287.06	25.5	35.87
Peonidin 3-*O*-Glucoside	463.12	301.07	23.1	34.03
Unidentified Peonidin	635.13	301.07	34.6	11.79
Cyanidin (dimalonyl)hexoside	621.11	287.06	29.6	9.08
Peonidin (malonyl)hexoside	549.12	301.07	27.9	7.85
Cyanidin (sinapoyl)hexoside	655.17	287.06	37.7	3.41
Unidentified Cyanidin	625.18	287.05	38.3	<LOQ
**Violet grain RMu13, 281.46 mg/kg**				
**Cyanidin Rutinoside**	595.17	287.05, 449.11	20.6	101.36
Peonidin Rutinoside	609.18	301.07, 463.12	24.6	52.92
Cyanidin (malonyl)hexoside	535.11	287.06	25.0	37.48
Cyanidin 3-*O*-Glucoside	449.11	287.06	18.7	27.07
Peonidin (malonyl)hexoside	549.12	301.07	29.1	26.13
Peonidin 3-*O*-Glucoside	463.12	301.07	23.0	21.45
Cyanidin (dimalonyl)hexoside	621.11	287.06	28.9	9.11
Peonidin (dimalonyl)hexoside	635.13	301.07	33.1	5.94
Pelargonidin Rutinoside	579.17	271.06, 443.10	22.8	<LOQ
Cyanidin (sinapoyl)hexoside	655.17	287.06	38.3	<LOQ
**Violet grain GC-14, 1.63 mg/kg**				
**Peonidin 3-*O*-Glucoside**	463.12	301.07	22.8	1.33
Peonidin (malonyl)hexoside	549.12	301.07	29.3	0.30
Malvidin 3-*O*-Galactoside	496.74	331.08	22.9	<LOQ
Peonidin Rutinoside	609.18	301.07, 463.12	24.6	<LOQ
**Violet grain GC-14/1, 0.11 mg/kg**				
**Peonidin 3-*O*-Glucoside**	463.12	301.07	23.4	0.11
**Violet grain GC-14/2, 0.2 mg/kg**				
**Cyanidin Rutinoside**	595.17	287.05, 449.11	20.6	0.10
**Peonidin 3-*O*-Glucoside**	463.12	301.07	23.2	0.10
Peonidin Rutinoside	609.19	301.07, 463.12	25.0	<LOQ
Cyanidin 3-*O*-Glucoside	449.11	287.05	17.8	<LOQ

^1^ Semi-bold font denotes the major anthocyanins in sample; ^2^ LOQ—values are lower than the limit of quantification (SN < 10), but higher than the limit of detection (SN > 3); ^3^ Content calculated as cyanidin equivalent.

**Table 2:** Quantification of anthocyanins by spectrophotometric pH—differential method.

Accession	Major Anthocyanin	λ_vis-max_ (nm) ^1^	ε	MW	Content mg/kg of Dry Matter
S10	Cyanidin (malonyl)glucoside	528	32,360	535.11	25.33
RMu13	Cyanidin Rutinoside	510	7000	595.17	328.2
RMu12	Peonidin Rutinoside	512	14,100	609.18	451.14

^1^ Maximum absorption in the visible region (400–700 nm).

**Table 3:** Plant material used in the work.

Characteristic	Accession ^1^
Lacking anthocyanin	Esto (vi1), GC-22yellow (vi2), GC-23 (vi3), GC-149 (vi4), GC-151 (vi5), GC-150 (vi6)
Yellow-seeded	GC-12, L2, L5, L7 (c)
Brown-seeded	RMu1 brown (vi1, Vs?), GC-22 brown (vi2, Vs?), GC-37(vi1, Vs)
Green-seeded	L8, L87, L301, RMu5, RMu1 green (C)
Violet-seeded	GC-14, GC-14/1, GC-14/2, RMu12, RMu13, S10 (Vs)

^1^ Identified or assumed genes are shown in brackets [16].

## Supplementary Materials

Supplementary materials are available online.
