# Supplementary material for: Anthocyanin Composition and Content in Rye Plants with Different Grain Color
Source: Molecules. 2018 Apr 19;23(4):948. doi: 10.3390/molecules23040948 (PMC6017340; doi:10.3390/molecules23040948)
Supplement: Supplementary file 1 [file molecules-23-00948-s001.pdf]

Formula	RT	Monoisotopic mass
C42H47O22		903.2559
C32H31O15	37.55	655.1663
C36H37O19		773.1929
C43H49O24		949.2614
C27H29O17		625.1405
C27H25O17	29.05	621.1092
C36H37O18		757.198
C23H23O12		491.119
C30H27O14		611.1401
C25H20O17		592.07
C30H33O19		697.1616
C24H23O14	25.03	535.1088
C30H31O20		711.1409
C30H27O13	33.46	595.1452
C25H25O14	30.1	549.1244
C20H19O10	18.58	419.0978
C20H19O10	22.41	419.0978
C36H37O18		757.198
C26H29O15		581.1506
C33H41O21		773.214
C31H29O14		625.1557
C21H21O11	16.13	449.3848
C21H21O11	18.2	449.3848
C33H41O20		757.2191
C21H21O10		433.1135
C27H31O15	20.65	595.1663
C33H41O20		757.2191
C32H39O20		743.2035
C27H31O16		611.1612
C32H39O19		727.2086
C27H31O16		611.1612
C32H39O20		743.2035
C15H11O6	23.72	287.0556
C42H47O23		919.2508
C36H37O20		789.1878
C27H25O18		637.1041
C36H37O19		773.1929
C23H23O13		507.1139
C30H27O15		627.135
C30H33O20		713.1565
C24H23O15		551.1037
C30H27O14	30.89	611.1401
C20H19O11	16.64	435.0927

C36H37O19		773.1929
C26H29O16		597.1456
C33H41O22		789.2089
C31H29O15		641.1506
C21H21O12	14.31	465.1033
C21H21O12	15.51	465.1033
C27H31O17		627.1561
C33H41O21		773.214
C21H21O11		449.1084
C27H31O16	17.83	611.1612
C26H29O16	15.29	597.1456
C32H39O21		759.1984
C27H31O17		627.1561
C27H31O17		627.1561
C32H39O21		759.1984
C15H11O7		303.0505
C44H51O23		947.2821
C25H27O13	31.51	535.4741
C25H27O13	34.53	535.1452
C32H31O15		655.1663
C32H31O14		639.1714
C22H23O11	29.32	463.124
C28H33O16		625.1769
C23H25O12	23.2	493.1346
C23H25O12	24.36	493.1346
C29H35O16		639.1925
C29H35O17		655.1874
C17H15O7		331.0818
C36H37O18		757.198
C27H25O16		605.1143
C30H27O13		595.1452
C24H23O13		519.1139
C30H27O12		579.1503
C25H25O13		533.1295
C20H19O9	20.59	403.1029
C36H37O17		741.2031
C26H29O14		565.1557
C21H21O10	18.33	433.1135
C33H41O19		741.2242
C27H31O14	22.78	579.1714
C33H41O19		741.2242
C26H29O14		565.1557
C32H39O19		727.2086
C27H31O15		595.1663

C27H31O15		595.1663
C32H39O19		727.2086
C15H11O5		271.0606
C43H49O22		917.2715
C28H27O17	33.32	635.1248
C24H25O12	33.21	505.1346
C31H29O14		625.1557
C25H25O14	28.5	549.1244
C31H29O13	39.99	609.1608
C21H21O10	23.35	433.1135
C34H43O21		787.2297
C22H23O11	20.97	463.124
C22H23O11	22.54	463.124
C28H33O15	24.6	609.1819
C27H31O15		595.1663
C33H41O20		757.2191
C28H33O16		625.1769
C28H33O16		625.1769
C16H13O6		301.0712
C43H49O23		933.2665
C24H25O13	30.57	521.1295
C31H29O14	37.16	625.1557
C21H21O11	21.34	449.1084
C21H21O11	24.99	449.1084
C27H31O16		611.1612
C22H23O12	19.02	479.119
C22H23O12	20.28	479.119
C22H23O10		447.1291
C27H31O15		595.1663
C27H31O16		611.1612
C28H33O17		641.1718
C16H13O7		317.0661

Compound Name
Cyanidin (coumaroyl)rutinoside hexoside
Cyanidin (sinapoyl)hexoside
Cyanidin (caffeoyl)hexoside hexoside
Cyanidin (caffeoyl)hexoside dihexoside
Cyanidin (glucuronosyl)hexoside
Cyanidin (dimalonyl)glucoside
Cyanidin coumaroylhexoside hexoside
Cyanidin (acetyl)hexoside
Cyanidin (caffeoyl)hexoside
Cyanidin (dioxalyl)hexoside
Cyanidin (malonyl-hexosyl)hexoside
Cyanidin (malonyl)hexoside
Cyanidin (malonyl-glucuronyl)hexoside
Cyanidin (coumaroyl)hexoside
Cyanidin (succinyl)hexoside
Cyanidin 3-O-arabinoside
Cyanidin pentoside
Cyanidin (coumaroyl)hexoside hexoside
Cyanidin sambubioside
Cyanidin dihexoside hexoside
Cyanidin (feruloyl)hexoside
Cyanidin 3-O-galactoside
Cyanidin 3-O-glucoside
Cyanidin hexosyl-rutinoside
Cyanidin deoxy-hexoside
Cyanidin rutinoside
Cyanidin rutinoside hexoside
Cyanidin sambubioside hexoside
Cyanidin sophoroside
Cyanidin (pentosyl)rutinoside
Cyanidin dihexoside
Cyanidin hexoside sambubioside
Cyanidin aglycone
Delphinidin (coumaroyl)rutinoside hexoside
Delphinidin (caffeoyl)hexoside hexoside
Delphinidin (dimalonyl)hexoside
Delphinidin (coumaroyl)hexoside hexoside
Delphinidin (acetyl)hexoside
Delphinidin (caffeoyl)hexoside
Delphinidin (malonyl-hexosyl)hexoside
Delphinidin (malonyl)hexoside
Delphinidin(coumaroyl)hexoside
Delphinidin 3-O-arabinoside

Delphinidin (coumaroyl)hexoside hexoside
Delphinidin sambubioside
Delphinidin dihexoside hexoside
Delphinidin (feruloyl)hexoside
Delphinidin 3-O-galactoside
Delphinidin 3-O-glucoside
Delphinidin (hexosyl)hexoside
Delphinidin (hexosyl)rutinoside
Delphinidin deoxy-hexoside
Delphinidin rutinoside
Delphinidin sambubioside
Delphinidin sambubioside hexoside
Delphinidin sophoroside
Delphinidin dihexoside
Delphinidin hexoside sambubioside
Delphinidin aglycone
Malvidin (coumaroyl)rutinoside hexoside
Malvidin (acetyl)galactoside
Malvidin (acetyl)glucoside
Malvidin (caffeoyl)hexoside
Malvidin (coumaroyl)hexoside
Malvidin 3-O-arabinoside
Malvidin sambubioside
Malvidin 3-O-galactoside
Malvidin 3-O-glucoside
Malvidin rutinoside
Malvidin dihexoside
Malvidin aglycone
Pelargonidin (caffeoyl)hexoside hexoside
Pelargonidin (dimalonyl)hexoside
Pelargonidin (caffeoyl)hexoside
Pelargonidin (malonyl)hexoside
Pelargonidin (coumaroyl)-hexoside
Pelargonidin (succinyl)-hexoside
Pelargonidin pentoside
Pelargonidin hexoside (coumaroyl)hexoside
Pelargonidin sambubioside
Pelargonidin hexoside
Pelargonidin (hexosyl)rutinoside
Pelargonidin rutinoside
Pelargonidin rutinoside hexoside
Pelargonidin sambubioside
Pelargonidin sambubioside hexoside
Pelargonidin sophoroside

Pelargonidin dihexoside
Pelargonidin hexoside sambubioside
Pelargonidin aglycone
Peonidin (coumaroyl)rutinoside hexoside
Peonidin (dimalonyl)hexoside
Peonidin (acetyl)hexoside
Peonidin (caffeoyl)hexoside
Peonidin (malonyl)hexoside
Peonidin (coumaroyl)hexoside
Peonidin 3-O-arabinoside
Peonidin dihexoside hexoside
Peonidin 3-O-galactoside
Peonidin 3-O-glucoside
Peonidin rutinoside
Peonidin sambubioside
Peonidin sambubioside hexoside
Peonidin sophoroside
Peonidin dihexoside
Peonidin aglycone
Petunidin (coumaroyl)rutinoside hexoside
Petunidin (acetyl)hexoside
Petunidin (coumaroyl)glucoside
Petunidin 3-O-arabinoside
Petunidin pentoside
Petunidin sambubioside
Petunidin 3-O-galactoside
Petunidin 3-O-glucoside
Petunidin deoxy-hexoside
Petunidin rutinoside
Petunidin sambubioside
Petunidin sophoroside
Petunidin aglycone

Comment (Aglyc	Comment (Ad	Comment (standard)
287.055023		
287.055023		
287.055023		
287.055023		
287.055023		
287.055023		
287.055023		
287.055023		
287.055023		
287.055023		
287.055023		
287.055023		
287.055023		
287.055023		
287.055023		
287.055023		blueberry
287.055023		
287.055023		
287.055023		
287.055023		
287.055023		
287.055023		blueberry
287.055023		blueberry
287.055023		
287.055023		
287.055023	449.11	
287.055023		
287.055023		
287.055023		
287.055023		
287.055023		
287.055023		
287.055023		blueberry, Sigma-Aldrich #79
303.049927		
303.049927		
303.049927		
303.049927		
303.049927		
303.049927		
303.049927		
303.049927		
303.049927		
303.049927		blueberry

303.049927		
303.049927		
303.049927		
303.049927		
303.049927		blueberry
303.049927		blueberry
303.049927		
303.049927		
303.049927		
303.049927	465.11	
303.049927		
303.049927		
303.049927		
303.049927		
303.049927		
303.049927		blueberry
331.081238		
331.081238		
331.081238		
331.081238		
331.081238		
331.081238		blueberry
331.081238		
331.081238		blueberry, Sigma-Aldrich #79[illegible]
331.081238		blueberry
331.081238		
331.081238		
331.081238		blueberry
271.060089		
271.060089		
271.060089		
271.060089		
271.060089		
271.060089		
271.060089		
271.060089		
271.060089		
271.060089		
271.060089		
271.060089	443.1	
271.060089		
271.060089		
271.060089		
271.060089		

271.060089		
271.060089		
271.060089		
301.070679		
301.070679		
301.070679		
301.070679		
301.070679		
301.070679		
301.070679		blueberry
301.070679		
301.070679		blueberry
301.070679		blueberry
301.070679	463.12	
301.070679		
301.070679		
301.070679		
301.070679		
301.070679		blueberry
317.065582		
317.065582		
317.065582		
317.065582		blueberry
317.065582		
317.065582		
317.065582		blueberry
317.065582		blueberry
317.065582		
317.065582		
317.065582		
317.065582		
317.065582		blueberry

457

311
